# Evaluation of Multiple Sclerosis Care and Its Impact on Brain Health: A United Arab Emirates Center Experience

**DOI:** 10.7759/cureus.81201

**Published:** 2025-03-25

**Authors:** Ruqqia Mir, Fatema Al-Kaabi, Yasmin Mitwally, Surangi Jayakody

**Affiliations:** 1 Consultant Neurology, Abu Dhabi Stem Cells Center (ADSCC), Abu Dhabi, ARE; 2 Hematology, Abu Dhabi Stem Cells Center (ADSCC), Abu Dhabi, ARE; 3 Public Health, National Multiple Sclerosis Society, Abu Dhabi, ARE; 4 Public Health, University of Warwick, Coventry, GBR

**Keywords:** brain health, multiple sclerosis, policy recommendations, public health, united arab emirates

## Abstract

Background: Multiple sclerosis is a chronic, complex neurological disease, often presenting with both visible and non-visible symptoms that significantly affect daily functioning. A holistic approach to multiple sclerosis care is essential for patient well-being, particularly in the unique sociocultural context of the United Arab Emirates, where factors such as insurance coverage and specialist availability can present additional challenges.

Methodology: A retrospective, service-improvement study of 81 individuals with multiple sclerosis was conducted at a specialized center in the United Arab Emirates to evaluate demographics, disease management practices, and support services. Data were extracted from electronic medical records covering the period from January 1, 2022, to January 1, 2024, and assessed against established quality measures, including crucial screening procedures recommended by the American Academy of Neurology’s Quality Measurement Set.

Results: Significant gaps were identified in essential multiple sclerosis care, particularly in consultations about employment challenges, sexual dysfunction screening, and cognitive function assessment. Of the 81 participants, 43 (53.09%) were women, and 38 (46.91%) were men, with a mean age of 40.09 years. Analysis of the Expanded Disability Status Scale scores showed no significant association with blood pressure (P=0.1067) or glycemic status (P=0.3891), but a positive correlation with age (P=0.0099).

Conclusion: An integrated, multidisciplinary model of multiple sclerosis care is crucial, emphasizing regular assessments of cognitive function, fatigue, sexual health, and work-related issues to optimize patient outcomes and enhance overall brain health in the United Arab Emirates. Additionally, systematic policy interventions that mandate comprehensive screening protocols could further strengthen care delivery. Such measures may foster earlier detection of hidden disabilities and better coordination among diverse healthcare professionals, ultimately improving the quality of life for individuals with multiple sclerosis.

## Introduction

Multiple sclerosis (MS) is characterized by demyelination in the central nervous system (CNS) and has profound effects on physical, cognitive, and emotional well-being, particularly in young adults, ultimately impacting career, personal relationships, and overall quality of life [1,2]. Existing evidence suggests a potential correlation between the effects of social determinants of health and the health outcomes of people with MS [3]. Thus, understanding local demographics is crucial for tailoring effective MS care within the United Arab Emirates (UAE) by addressing the neurological, psychological, social, and environmental factors that affect patients’ daily lives [3]. These factors are critical for understanding the disease burden and optimizing effective care strategies within the region [4].

Recent quality improvement initiatives emphasize comprehensive monitoring practices that focus on both physical and psychosocial dimensions. The American Academy of Neurology’s (AAN) Quality Measurement Set 2020 outlines crucial monitoring practices [5], including timely magnetic resonance imaging (MRI) reviews, disease-modifying therapy (DMT) checks, and assessments for bladder, bowel, and sexual dysfunction, as well as cognitive impairment, fatigue, exercise, and physical therapy. These measures are vital for reducing disability and maintaining quality of life [5].

Non-motor symptoms such as cognitive impairment, fatigue, and sexual dysfunction significantly affect patient well-being, yet are often underreported and underassessed. Sexual dysfunction is similarly widespread, affecting up to 70% of individuals, yet it can occur even in the absence of severe disability [6]. The degree of disability in MS is commonly measured with the Expanded Disability Status Scale (EDSS), which focuses heavily on ambulation and may not fully capture these nonphysical symptoms [6].

MS also poses a substantial economic burden on healthcare systems due to high medication costs, productivity loss, and caregiver strain [7]. Patients with advanced MS utilize greater resources (hospitalizations, tests, and scans) and often incur costs linked to comorbidities and overall productivity loss [8].

Cognitive impairment affects 40-60% of people with MS, impacting attention, memory, and executive function [9]. Fatigue is one of the most reported symptoms, affecting up to 50-90% of patients at some point [10]. Both of these symptoms can have a significant impact on quality of life. Public health initiatives should focus on holistic interventions such as improving mental health awareness, earlier identification, and provision of resources for affected individuals, which is crucial for tailored support and interventions [11]. Additionally, modifiable brain health monitoring variables such as monitoring of body mass index (BMI), blood pressure, glycemic status, and vitamin D levels are essential components of comprehensive brain health care [12]. Screening and consultations regarding employment and fall risk are also vital elements to help address the broader societal challenges faced by MS patients [13].

Implications related to poor access to rehabilitation interventions due to a lack of specialists and limited insurance coverage can negatively affect people with MS within the UAE region, as it can pose challenges in fully understanding the dysfunction of MS patients and standardizing personalized clinical protocols for treating these variables associated with fatigue, cognition, and work-related difficulties [14]. Given the complex nature of MS and the myriad factors that affect disease progression and patient outcomes, this study aimed to systematically evaluate the current status of MS care at a specialized center in the UAE. Specifically, we sought to determine the extent to which demographic, clinical, and support-service elements, such as cognitive, sexual, and employment-related aspects, were addressed, thus identifying gaps in care and proposing evidence-based recommendations to enhance brain health and quality of life in this population.

## Materials and methods

Study design and population

This study followed a quality improvement (QI) framework, focusing on care assessment against established standards from the AAN Quality Measurement Set 2020 [5]. Although the framework was QI-oriented, the design was fundamentally observational, examining existing clinical data to identify care gaps. Initially, 110 patients were considered; however, only 81 participants aged 18 to 65 met the inclusion criteria, with data gathered from the electronic medical records. Pregnant women and those participating exclusively in telemedicine consultations were excluded.

Data collection

Demographic, clinical, and support service data were extracted and analyzed to identify gaps in patient care covering the period from January 1, 2022, to January 1, 2024. The variables collected for analysis included demographic variables such as gender, age, nationality (Emirati vs. non-Emirati), and socioeconomic status as perceived by their insurance level; disease management variables including the type of MS, monitoring practices (MRI, DMTs, fatigue, cognitive function, physical activity), and health parameters (BMI, blood pressure, glycaemic status, vitamin D levels); and support services variables, which encompassed mental health assessments, sexual dysfunction screening, employment status, and consultations related to falls risk. For cognitive assessment, the Symbol Digit Modalities Test (SDMT) or Montreal Cognitive Assessment (MoCA) was used, and fatigue was measured via the Modified Fatigue Impact Scale (MFIS) or documented during EDSS evaluation. All data were anonymized and stored in a secure database to ensure patient confidentiality.

Data analysis

Data analysis was conducted using Minitab v17, employing descriptive statistics to summarize the data and comparative analysis to identify gaps in care. In the descriptive analysis, counts and percentages were calculated for categorical variables (such as gender, nationality, type of MS, monitoring practices, and support services) to provide an overview of their distribution within the study population. Additionally, for continuous variables such as age, BMI, and hemoglobin A1C (HbA1C) levels, the mean and standard deviation were calculated to summarize central tendency and variability. In the comparative analysis, the relationship between demographic variables and disease management practices was assessed to identify any significant patterns or differences in care delivery between different population groups. Given the primary aim of service assessment rather than inference of causality, no formal adjustments for potential confounding factors (e.g., age, gender, insurance coverage) were performed in this analysis.

Ethical considerations

This study was conducted following institutional and international guidelines for human subjects research. Informed consent was obtained from all participants at the time of their initial consultation, allowing for the use of their anonymized data in quality improvement and research activities. The study's primary aim was to enhance service delivery and optimize patient outcomes, consistent with the approved ethical framework.

## Results

This center specializes in receiving people with MS and consists of two specialized consultant neurologists, one general practitioner, and a specialized MS nurse. There is access to MS specialized physical therapists and other specialties like urologists, gastroenterologists, internal medicine, and cardiologists. During this study, there was limited access to in-house psychologists or trained psychiatrists. Although rehabilitation services are not available in the Centre, some patients have access to other facilities to avail themselves of this service.

Demographic characteristics

Of the 81 patients, 43 (53.09%) were female, 38 (46.91%) were male, with a mean age of 40.09 (±11.11) years. The population was diverse, comprising 36 (44.44%) Emiratis and 45 (55.56%) non-Emiratis. Regarding multiple sclerosis subtypes, 34 (41.98%) had relapsing-remitting MS (RRMS), 40 (49.37%) had secondary progressive MS (SPMS), six (7.41%) had primary progressive MS (PPMS), and one (1.23%) had radiologically isolated syndrome (RIS). A further overview of the study population is summarized in Table 1, providing an overview of the patient profile before exploring gaps in essential assessments and screenings, which are crucial for optimizing patient outcomes.

**Table 1:** Demographic Characteristics of the Study Population

Variable	Count (%)
Gender M/F	
Female	43 (53.09%)
Male	38 (46.91%)
Age, mean (Std dev)	40.09 (11.11)
BMI, mean (Std dev)	27.74 (6.04)
Emirati Yes/No	
No	45 (55.56%)
Yes	36 (44.44%)
Type of MS (Multiple Sclerosis)	
RRMS (Relapsing Remitting MS)	34 (41.98%)
SPMS (Secondary Progressive MS)	40 (49.37%)
PPMS (Primary Progressive MS)	6 (7.41%)
RIS (Radiologically Isolated Syndrome )	1 (1.23%)

Critical gaps in care

**Figure 1:**
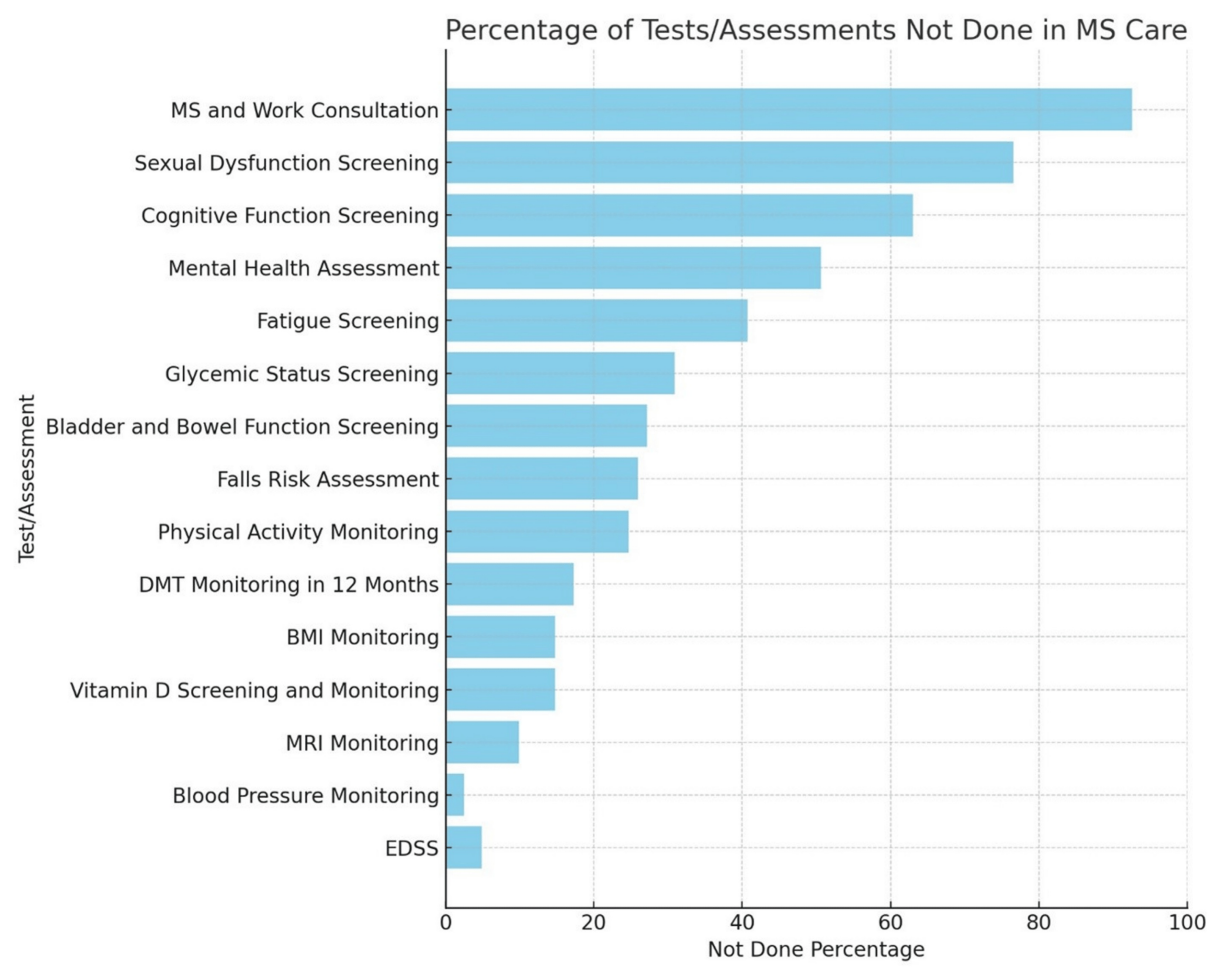
Bar Chart Showing the "Not Done" Assessments in Descending Order MS: multiple sclerosis, BMI: Body Mass Index, DMT: disease-modifying therapy, EDSS: Expanded Disability Status Scale

Analysis revealed significant "Not Done" instances across various critical care components, with MS and Work Consultation being notably absent in 75 (92.59%). Sexual Dysfunction Screening was also largely overlooked, with 62 (76.54%) of patients not receiving this assessment. Cognitive Function and fatigue screening was another area of concern, with 51 (62.96%) of patients not being evaluated. Cognition was assessed either using the SDMT or MoCA. Fatigue assessment was done either during the EDSS evaluation or by using the MFIS. The bladder, bowel, and sexual dysfunction were identified during the EDSS evaluation only, and if necessary, referrals to appropriate specialties were made. These findings are summarized in Table 2 and visually represented in Figure 1.

**Table 2:** Summary of "Not Done" Assessments and Screenings in Multiple Sclerosis (MS) Care

Test/Assessment	Not Done Count (%)
MS and Work Consultation	75 (92.59%)
Sexual Dysfunction Screening	62 (76.54%)
Cognitive Function Screening	51 (62.96%)
Mental Health Assessment	41 (50.62%)
Fatigue Screening	33 (40.74%)
Glycaemic Status Screening	25 (30.86%)
Bladder and Bowel Function Screening	22 (27.16%)
Falls Risk Assessment	21 (25.93%)
Physical Activity Monitoring	20 (24.69%)
DMT (Disease-Modifying Therapy) Monitoring in 12 Months	14 (17.28%)
BMI Monitoring (Body Mass Index)	12 (14.81%)
Vitamin D Screening and Monitoring	12 (14.81%)
MRI Monitoring	8 (9.88%)
Blood Pressure Monitoring	2 (2.47%)
EDSS (Expanded Disability Status Scale )	4 (4.9%)

Health parameter analysis

No significant association was found between the EDSS scores and various health parameters, including blood pressure and glycaemic status.

**Figure 2:**
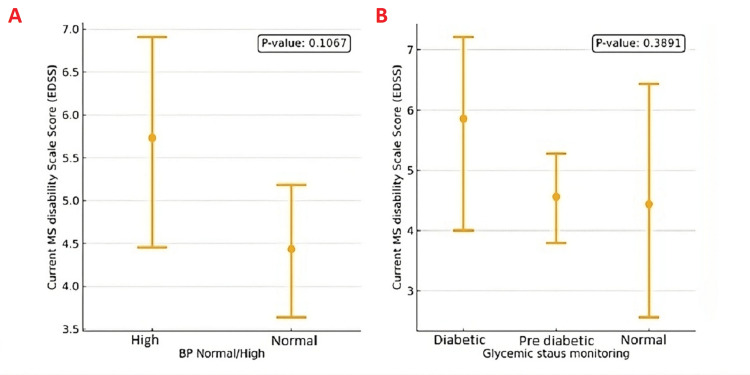
Interval Plots of EDSS Scores vs. Blood Pressure and Glycemic Status (A) EDSS scores among patients with normal vs. high blood pressure (P = 0.1067). (B) EDSS scores among diabetic, pre‐diabetic, and normal glycemic status groups (P = 0.3891). MS: multiple sclerosis, EDSS: Expanded Disability Status Scale; BP: blood pressure

However, older age showed a positive correlation with disability scores. The mean EDSS score for patients with normal blood pressure was lower (4.5) compared to those with high blood pressure (6.0), although this difference was not statistically significant (P-value: 0.1067). Similarly, patients who had their glycaemic status monitored did not show a statistically significant difference in EDSS scores compared to those with prediabetes or diabetes (P-value: 0.3891). These analyses were exploratory, reflecting an observational approach aimed at highlighting potential trends. The results are depicted in Figure 2.

**Figure 3:**
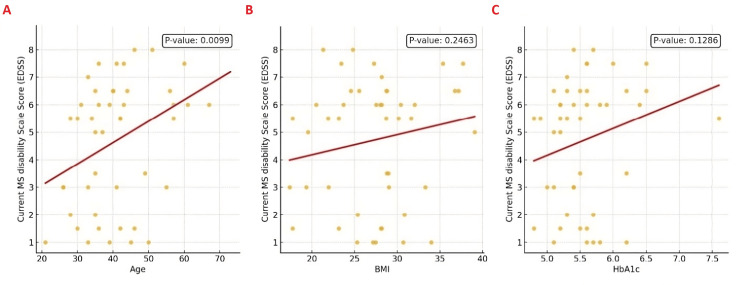
Regression Plots Showing the Correlation of EDSS With Age, BMI, and HbA1c (A) EDSS vs. Age (R = 0.35, P = 0.0099). (B) EDSS vs. BMI (R = 0.15, P = 0.2463). (C) EDSS vs. HbA1c (R = 0.20, P = 0.1286). MS: multiple sclerosis, EDSS: Expanded Disability Status Scale; BMI: Body Mass Index; HbA1c: Hemoglobin A1c

Further correlation analysis between EDSS scores and age, BMI, and HbA1c levels provided additional insights. We found that the correlation coefficient for age was R=0.35 (P=0.0099), indicating a notable positive correlation, while the correlation coefficients for BMI and HbA1c were R=0.15 (P=0.2463) and R=0.20 (P=0.1286), respectively, indicating that these trends did not reach statistical significance. All correlations serve to illustrate general associations rather than establish definitive causal links. This suggests that older patients tended to have higher disability scores, but that any mild trend toward higher disability with increasing BMI or poorer glycaemic control was not statistically meaningful in this cohort. The correlations are illustrated in Figure 3.

**Figure 4:**
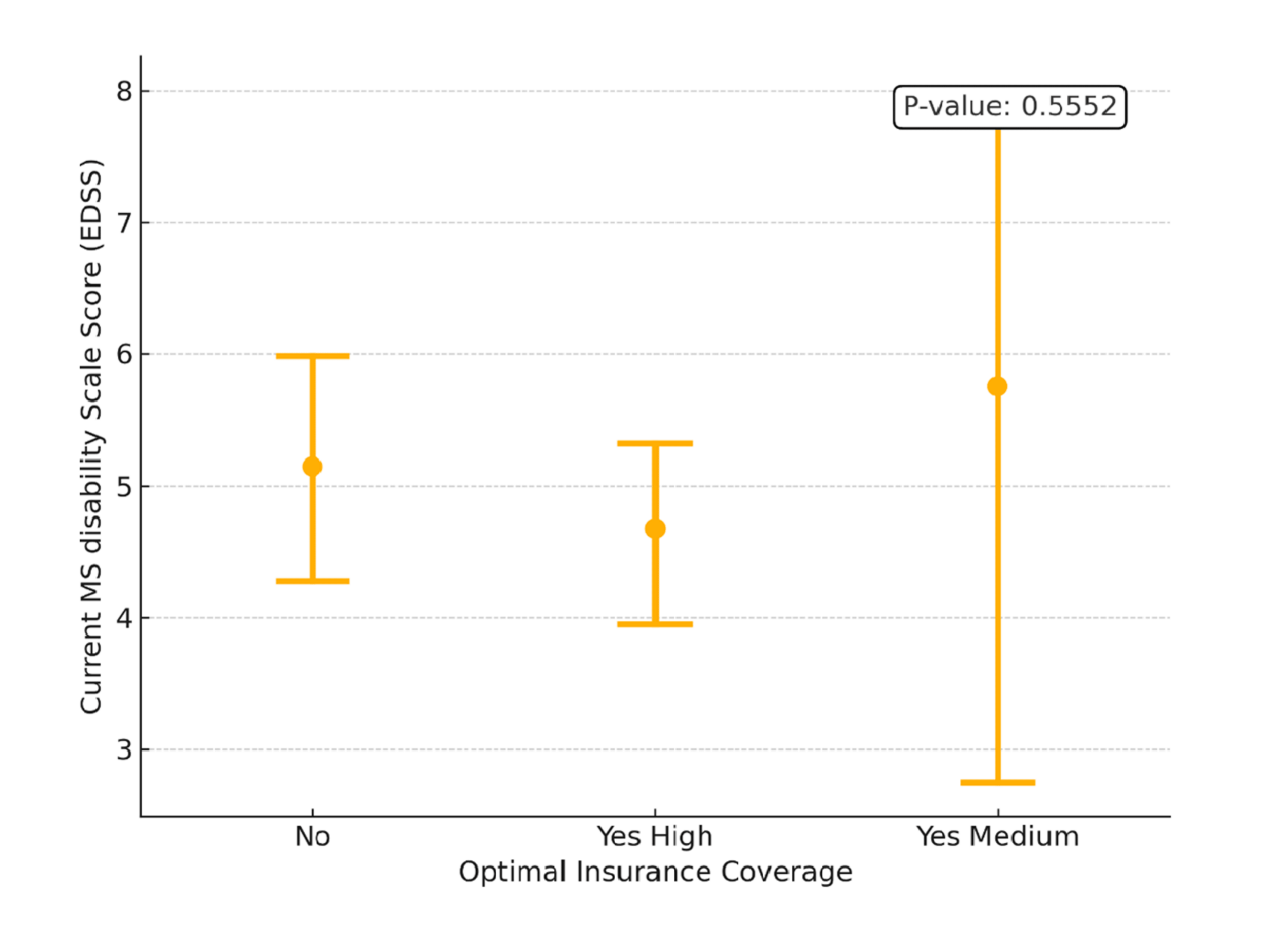
Interval Plot of EDSS Scores vs. Optimal Insurance Coverage MS: multiple sclerosis, EDSS: Expanded Disability Status Scale

Further analysis explored the relationship between the EDSS scores and the level of optimal insurance coverage, which can correlate with their financial and socioeconomic status. As shown in Figure 4, patients with no optimal insurance coverage or lower socioeconomic status had a mean EDSS score of approximately 6.0, while those with high insurance coverage and, thus, higher socioeconomic status had a lower mean EDSS score of around 4.5. Interestingly, patients with medium-level insurance coverage exhibited a wide range of EDSS scores, though the average was like that of the high coverage group. However, the differences in EDSS scores across these insurance categories were not statistically significant (P-value: 0.5552). While coverage level offers some insight into socioeconomic factors, other real-world elements (e.g., clinical history, psychosocial support) may also influence disability outcomes.

## Discussion

Advances in MS care have led to quality improvement strategies earlier in the disease onset, promoting quality of life and enhancing medical treatment outcomes. In line with our stated objective of evaluating the comprehensiveness of MS care -particularly regarding cognitive, sexual, and employment-related aspects - we identified several key findings that underscore existing care gaps.

Significant findings

The evaluation revealed critical gaps in MS care, particularly regarding underassessed dimensions such as sexual health, cognitive function, and employment-related issues. Some aspects of care, such as monitoring of DMTs and MRI scans, were adequately addressed. Significant deficiencies were noted in areas crucial to overall patient well-being, particularly in addressing hidden neurological disabilities that impact quality of life, highlighting the unperceived societal barriers and discrimination that people with MS may face [15]. Females made up just over half of our cohort, which aligns with broader epidemiological data indicating a higher prevalence of MS in women worldwide.

These gaps underscore the need for an integrated, multidisciplinary approach to MS care that includes regular assessments beyond visible neurological symptoms, as there is little known about incidence and prevalence of such comorbidities in people with MS in this region [16]. A recent study showed that hidden disabilities are common and may remain unchanged or minimally improved after the immediate post-diagnostic or relapse period [17]. The non-visible aspects of the lives of people with MS need to be observed and expressed by their symptoms, as well as by understanding the societal barriers and discriminations they may face [15]. This includes addressing social determinants of health - that is, the “risks of risks” - on health in most chronic neurological diseases [3]. High “Not Done” rates for key non-motor issues, such as sexual dysfunction and cognitive assessments, further emphasize how subtle, yet impactful, manifestations of MS can be overlooked without systematic screening.

Identified barriers

Barriers to effective care included cultural perceptions, insufficient resources, and lack of provider awareness of these essential assessments. Employment considerations are crucial for maintaining patients' financial independence and social integration yet were largely neglected in this cohort [18]. Research around employment discrimination is limited within the region, prompting the need for future research to be inclusive of societal barriers and non-medical factors that influence health outcomes.

Integration of findings into the literature

The results of this study are consistent with the broader literature on MS care, which highlights the need for a holistic approach that encompasses physical, cognitive, and emotional health. Comprehensive MS care has been shown to improve patient outcomes by addressing a wide range of factors that influence disease progression and quality of life [19]. Our findings suggest that disparities in MS care may be influenced by both financial and racial/ethnic backgrounds, such as the distinction between Emirati and non-Emirati patients, as well as variable insurance coverage, mirroring global trends in healthcare inequities [3]. However, as evident in this study, there is often a gap between theoretical models of comprehensive care and their implementation in clinical practice [20]. The findings align with previous research that underscores the variability in care practices across different regions and healthcare settings. This suggests that taking into consideration local factors, such as resource availability, cultural attitudes, and meaningful interventions targeting social determinants of health, would significantly influence the delivery of care [3].

Public health implications

The health system implications of this study are far-reaching for the management of MS in the UAE and similar settings. The identified gaps in managing MS suggest significant challenges for the healthcare system that could result in delayed interventions and result in increased utilization of healthcare resources in the long run.

The findings suggest the need for a more structured and standardized approach to comprehensive MS management by the healthcare providers by assessing not only the clinical needs but also the individuals’ factors of social determinants of health such as gender, race and ethnicity impacting both the visible and non-visible symptoms of the disease, as well as the impact of societal barriers and discrimination to optimize care [15]. 

Sexual dysfunction, cognitive impairment, and fatigue are significant issues for individuals with MS. Healthcare providers should be trained and encouraged to incorporate regular assessments of cognitive function, fatigue screening, sexual health, and employment-related concerns into their care protocols [21]. All these factors could potentially address the social determinants of health that contribute to the overall MS prognosis [3].

Health equity and care access

MS is more common in women than men, with women reporting different characteristics such as more fatigue and anxiety and notably an increasing relationship of MS outcomes with race, ethnicity, socioeconomic status and stigmatization [3]. Special attention should be given to vulnerable populations such as expatriates, those with lower socioeconomic status who have an additional barrier to access comprehensive MS care. This study did not include “Young people with MS”, but similar guidelines should apply to young people and children with MS.

Health policy recommendations

Health policymakers in the UAE should consider introducing guidelines that mandate comprehensive care models, including assessments of both visible and non-visible symptoms as part of routine MS care. Such policies can recommend a whole system approach to MS care which includes healthcare worker capacity building, and involving the community and employers in health promotion activities on MS. This will enable us to change the view of a disabled persons from passive to active members of the society as per the 2006, Convention on the Rights of Persons with Disabilities [22].

Limitations and strengths

The retrospective nature of data collection might introduce biases and, along with the focus on a single centre, limits the study's generalizability; additional barriers related to access and socio-cultural attitudes were not studied, and variations in healthcare spending and resource allocation, as explored by Dieleman et al. (2020), may contribute to the discrepancies observed in care practices [23]. Yet despite these limitations, the study boasts several notable strengths: it utilizes a large and diverse patient population, which enhances the representativeness and relevance of its findings; it adopts a comprehensive approach that integrates clinical data, demographic information, and support service evaluations, thereby providing a multifaceted insight into MS care practices; and it employs rigorous analytical methods through both descriptive and comparative statistical analyses to thoroughly examine care gaps. Moreover, by focusing on a specialized UAE centre, the study offers unique, region-specific insights that can inform both local clinical practices and broader public health policies, and its adherence to ethical standards through proper data anonymization and informed consent further reinforces the robustness and integrity of the research.

## Conclusions

In conclusion, our findings reveal substantial gaps in comprehensive MS care at a specialized UAE center, particularly in the areas of cognitive and sexual health, as well as employment-related support. We recommend more structured policy measures such as: mandating routine assessments for cognitive, sexual, and vocational needs within national MS guidelines; forming multidisciplinary teams - including mental health professionals, physical therapists, and vocational counselors - across specialized centers; and expanding insurance coverage to ensure equitable access to necessary therapies and rehabilitation services. These strategies not only address the social determinants of health but also encourage earlier patient engagement, symptom self-reporting, and collaborative case management, ultimately enhancing the quality of life for individuals with MS in the UAE.
